# HAPPY: Hip Arthroscopy Portal Placement Using Augmented Reality

**DOI:** 10.3390/jimaging8110302

**Published:** 2022-11-06

**Authors:** Tianyu Song, Michael Sommersperger, The Anh Baran, Matthias Seibold, Nassir Navab

**Affiliations:** 1 Chair for Computer Aided Medical Procedures & Augmented Reality, Technical University Munich, 85748 Munich, Germany; 2 Laboratory for Computational Sensing and Robotics, Johns Hopkins University, Baltimore, MD 21205, USA; 3 Research in Orthopedic Computer Science, Balgrist University Hospital, University of Zurich, 8008 Zurich, Switzerland

**Keywords:** augmented reality, hip arthroscopy, endoscope placement

## Abstract

Correct positioning of the endoscope is crucial for successful hip arthroscopy. Only with adequate alignment can the anatomical target area be visualized and the procedure be successfully performed. Conventionally, surgeons rely on anatomical landmarks such as bone structure, and on intraoperative X-ray imaging, to correctly place the surgical trocar and insert the endoscope to gain access to the surgical site. One factor complicating the placement is deformable soft tissue, as it can obscure important anatomical landmarks. In addition, the commonly used endoscopes with an angled camera complicate hand–eye coordination and, thus, navigation to the target area. Adjusting for an incorrectly positioned endoscope prolongs surgery time, requires a further incision and increases the radiation exposure as well as the risk of infection. In this work, we propose an augmented reality system to support endoscope placement during arthroscopy. Our method comprises the augmentation of a tracked endoscope with a virtual augmented frustum to indicate the reachable working volume. This is further combined with an in situ visualization of the patient anatomy to improve perception of the target area. For this purpose, we highlight the anatomy that is visible in the endoscopic camera frustum and use an automatic colorization method to improve spatial perception. Our system was implemented and visualized on a head-mounted display. The results of our user study indicate the benefit of the proposed system compared to baseline positioning without additional support, such as an increased alignment speed, improved positioning error and reduced mental effort. The proposed approach might aid in the positioning of an angled endoscope, and may result in better access to the surgical area, reduced surgery time, less patient trauma, and less X-ray exposure during surgery.

## 1. Introduction

Arthroscopy is a mostly outpatient procedure used to diagnose or treat abnormalities and disorders in growing joints. It can enable the diagnosis and treatment of a broad variety of non-inflammatory, inflammatory, and infectious types of arthritis, as well as various injuries within the joint. To perform an arthroscopic procedure, orthopedic surgeons insert an arthroscope, a tube-like viewing instrument, through a small incision. The incision size depends on the size of the examined joint, but can typically be approximated by the size of a buttonhole. The arthroscope includes a small tube that contains optical fibers and lenses, which is connected to a video camera. In a typical setup, the camera view visualizing the interior of the examined joint is displayed on an external monitor.

Minimal access techniques through surgical trocars seek to perform surgical procedures while avoiding the morbidity associated with conventional surgical wounds [1]. Although hip arthroscopy is becoming more common for a growing array of indications due to improvements in instrumentation and more comfortable techniques, it still involves considerable challenges. Due to the hip joint’s overall structure and difficulties with direct access and visualization, several portal-placement-related complications can occur. In the presence of a greater soft-tissue envelope, patient positioning, portal placement, and instrument triangulation can all be significantly more difficult in obese people. The learning curve for hip arthroscopy among surgeons is typically referred to as “steep”, which means that the necessary skills and experience are difficult to attain while minimizing complications. These issues are associated with the challenges of the procedure, such as the limited maneuverability, the depth of the joint, and the distance of the hands from the point of the operating instruments [2]. In particular, the viewing direction of the endoscopic camera is often angled and not aligned with the direction of the instrument. Various conventional arthroscopic systems are angled by $30^{\circ }$ [3]. This drastically increases the complexity of the hand–eye coordination to navigate the endoscope to the target anatomy.

In this work, we propose an augmented reality (AR) system to support endoscope positioning for arthroscopy. Tracking the endoscopic instrument as well as the patient’s anatomy facilitates the augmentation of the angled endoscope frustum and in situ visualization of patient anatomy from pre-operative CT data. To evaluate the effectiveness of our system, we conduct a user study with a phantom setup, comparing the performance of our AR system to baseline insertions without additional support. Finally, we report and discuss our results, confirming the effectiveness of our AR system.

## 2. Related Work

To date, there are only a few works that integrate components related to our proposed system. To overcome the challenges of portal placement, Traub et al. [4] introduced an AR application to provide portal placement planning, but also in situ visualization for minimally invasive cardiovascular surgery. However, in their system, the portal placement planning was performed offline before the procedure and visualized on a see-through, 2D video screen.

In recent years, AR became more accessible with the useo f commercially available head-mounted displays (HMD). The applications for the use of AR include robotics, manufacturing, product assembly, medical training, and image-guided procedures [5,6,7,8]. Similar to the augmentation of the endoscope frustum in our work, Fotouhi et al. [9] introduced the concept of Interactive Flying Frustums, which can store the spatially aware surgical data for C-arm re-orientation and alignment. For each acquired X-ray, the frustum was augmented in space and the X-ray image was visualized in the frustum, allowing for a comparison of various C-arm poses. The proposed AR solution showed clear benefits of reducing the operating time and radiation dose while maintaining similar accuracy [10] for the K-wire insertion and the placement of the acetabular cup. In our system, we leveraged a similar frustum augmentation strategy. While, in [10], the augmentations were used to visualize X-ray images after acquisition with a C-arm, we used system for real-time endoscope navigation support with additional in situ visualization.

Furthermore, Qian et al. [11] proposed an AR assistance system for minimally invasive robotic surgery. To estimate the orientation and position of the endoscope, the camera frustum was augmented on an HMD and the surgical environment was reconstructed from a point cloud stream within the camera frustum.

To visualize anatomical structures inside the body in an in situ AR visualization, Bichlmeier et al. [12] proposed a method of contextual anatomic mimesis. The augmented anatomy, inside a focus area, was indicated by an outline, visually separating the real patient anatomy from the virtual content, where transparency levels are adjusted to support perceptual understanding. A similar approach was later integrated into intra-operative settings [13]. Spatial understanding and depth perception play an important role in AR applications for the correct perception of the relationship between real and virtual content. Many works [14,15,16,17] have addressed techniques that could further improve the spatial understanding of the virtual augmentations in an AR system. In other domains, only rendering virtual volumetric data, especially colorization in combination with shadowing, has been shown to provide useful perceptual cues for understanding depth [18,19].

In this paper, we propose a method that combines several state-of-the-art AR techniques to enhance hip arthroscopy and try to overcome the challenges presented in the current endoscope positioning procedure. To the best of our knowledge, this is the first time that an HMD-based AR prototype has been introduced for hip arthroscopy optimal endoscope positioning.

## 3. Methodology

This section describes the components of the proposed AR system. Those include the calibration of the endoscopic instrument, described in Section 3.1, the calibration between an external tracking system and the HMD space, described in Section 3.2, and the augmentation of the endoscope frustum, as well as the in situ visualization of the patient anatomy, described in Section 3.3 and Section 3.4.

### 3.1. Tool Calibration

Since the marker is rigidly attached to the endoscope, the calibration problem can be formulated as a pivot calibration. This system of equations can be solved if we rotate the endoscope around a fixed point and collect the tracking poses of the marker. For every tracked pose, the homogeneous rigid body transformation ${}^{\mathrm{tool}}T_{\mathrm{tracker}}^{}\left(i\right)$ is defined as:

(1) $${}^{\mathrm{tool}}T_{\mathrm{tracker}}^{}\left(i\right)=\left[\begin{array}{cc} R_{i} & t_{i}\\ 0^{⊤} & 1 \end{array}\right]$$

Therefore, for all transformations, we have the following formulation:

(2) $$\left[\begin{array}{c} p_{WCS}\\ 1 \end{array}\right]=\left[\begin{array}{cc} R_{i} & t_{i}\\ 0^{⊤} & 1 \end{array}\right]\left[\begin{array}{c} p_{TCS}\\ 1 \end{array}\right]\;$$

or

(3) $$R_{i}p_{TCS}-p_{WCS}=-t_{i}$$

where $p_{WCS}$ and $p_{TCS}$ are the translation from the tracker’s frame to the pivoting point and the translation from the marker to the tip. We can re-write and stack Equation (3) to form the overdetermined linear equation system:

(4) $$\left[\begin{array}{cc} R_{1} & -I\\ . & .\\ . & .\\ . & .\\ R_{n} & -I \end{array}\right]\left[\begin{array}{c} p_{TCS}\\ p_{WCS} \end{array}\right]=\left[\begin{array}{cc} -t_{1}\\ . & .\\ . & .\\ . & .\\ -t_{n} \end{array}\right]\;$$

Finally, to approximate the best solution, we adopted the least-squares minimization using Moore–Penrose inverse [20].

### 3.2. Tracking Space to HMD Space Calibration

**Figure 1:**
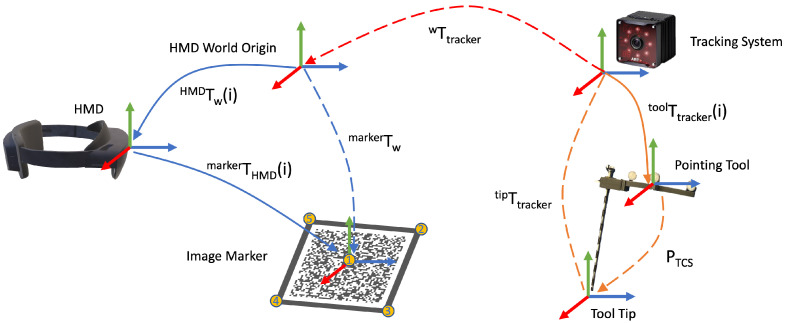
The calibration transformation chain. Transformations, shown as solid line arrows, are directly estimated, while transformations, shown as dash-line arrows, are derived. The blue color indicates the transformations in the HMD coordinate frame, and the orange color illustrates the transformations in the tracking system. The goal of tracking space to an HMD space calibration is to find the ${}^{W}T_{\mathrm{tracker}}^{}$, shown in red.

Due to the limitations of image-based tracking accuracy and tracking volume on HMD, an outside-in tracking system was employed. However, the introduction of an external tracking system brought a new coordinate frame to the setup. Therefore, a calibration procedure to register the HMD to the tracking system needed to be performed, as shown in Figure 1. We first defined five unique points $x_{\mathrm{HMD}}\left(1-5\right)$ on a image marker that can be tracked by the HMD. Then, we used a tool tracked by the tracking system to mark the same point set, so that they were in the tracker coordinate frame as $p_{\mathrm{tracker}}\left(1-5\right)$. Therefore, we could use the point cloud registration method to solve the transformation between two point sets, which essentially solves the following equation:

(5) $${}^{W}T_{\mathrm{tracker}}^{}=\underset{{}^{W}{\hat{T}}_{\mathrm{tracker}}}{\arg}\min\;\;d\;\left({\;}^{W}{\hat{T}}_{\mathrm{tracker}}p_{\mathrm{tracker}}\left(i\right),x_{\mathrm{HMD}}\left(i\right)\right)$$

where ${}^{W}T_{\mathrm{tracker}}^{}$ is the estimated transformation between two point sets, and the calibration matrix between the tracker and HMD space. To solve this transformation, we broke it down into a rotation component ${}^{W}R_{\mathrm{tracker}}$ and translation component ${}^{W}t_{\mathrm{tracker}}$ and estimated these sequentially. The detailed algorithm is demonstrated in Algorithm 1.

**Algorithm 1** Tracking Space to HMD Space Calibration $\bar{\left(.\right)}$: expected value **procedure**point sets registration(*M* pairs)         ▹ M = 5     $\bar{x_{\mathrm{HMD}}}=\frac{1}{M}\sum_{i=1}^{M}x_{\mathrm{HMD}}\left(i\right)$     $\bar{p_{\mathrm{tracker}}}=\frac{1}{M}\sum_{i=1}^{M}p_{\mathrm{tracker}}\left(i\right)$     **for** N = 1 to M **do**         $x_{\mathrm{HMD}}^{*}\left(N\right)=x_{\mathrm{HMD}}\left(N\right)-\bar{x_{\mathrm{HMD}}}$         $p_{\mathrm{tracker}}^{*}\left(N\right)=p_{\mathrm{tracker}}\left(N\right)-\bar{p_{\mathrm{tracker}}}$     **end for**     ${}^{W}R_{\mathrm{tracker}}=\mathrm{error}\sum_{i=1}^{M}{{(}^{W}R_{\mathrm{tracker}}p_{\mathrm{tracker}}^{*}\left(i\right)-x_{\mathrm{HMD}}^{*}\left(i\right))}^{2}$     ${}^{W}t_{\mathrm{tracker}}=\bar{x_{\mathrm{HMD}}}{-}^{W}R_{\mathrm{tracker}}\bar{p_{\mathrm{tracker}}}$     ${}^{W}T_{\mathrm{tracker}}^{}=\left[\begin{array}{ccc} {}^{W}R_{\mathrm{tracker}} & {}^{W}t_{\mathrm{tracker}}0^{⊤} & 1 \end{array}\right]$ **end procedure**


### 3.3. Frustum Generation and Endoscopic Streaming

**Figure 2:**
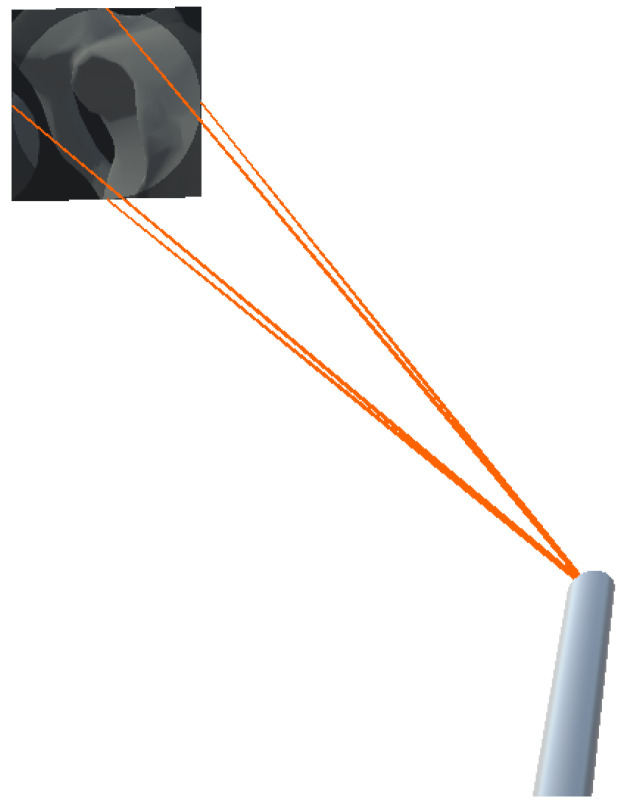
Visualization of the virtual frustum of the angled endoscope. The rendered image was projected onto the far clipping plane of the virtual camera.

To visualize the anatomy that is located along the view of the angled endoscope, we attached a virtual camera angled at 30 degrees to the tracked endoscope model. The camera view frustum was visualized based on the the standard 11 DoF camera parameters. The 6-DoF extrinsic parameters was calculated using the tracking information, and the 5-DoF intrinsic parameters were derived from the simulated virtual camera. We configured the virtual camera to only render the patient anatomy of the registered pre-operative data. To aid navigation of the endoscope, we augmented the camera frustum on the HMD, while the virtual camera’s view was projected onto its far clipping plane. This setup is illustrated in Figure 2. Additionally, the 512 × 512 endoscopic view was encoded and streamed to an external monitor via a TCP socket connection.

### 3.4. In-Situ Visualization

In order to provide a better spatial perception of the anatomical structures in the endoscope field of view, we augmented the part of the registered pre-operative anatomical bone model in the field of view of the endoscope. In an effort to reduce visual clutter in AR, the peripheral anatomy was rendered transparent, where transparency increased with increased distance to the focus area. To highlight the focused area containing the endoscopic camera frustum, we integrated an outline, adapted from the virtual window technique [12], for in situ visualization. We dynamically defined the size of the focus area based on the width of the frustum cone at the intersection with the virtual anatomical model. During rendering, we decided that if each vertex of the anatomical model was located within the cone frustum of the endoscopic camera, i.e., if the distance to the cone center line was smaller than the cone radius, then the cone radius at the depth of a given vertex position is defined by:

(6) $$r_{cone}=abs\left(\frac{r_{far}}{d_{far}\cdot d_{vertex}}\right)$$

where $r_{far}$ is the cone radius at the far clipping plane, $d_{far}$ is the distance between the virtual camera center and the far clipping plane along the cone center and $d_{vertex}$ is the distance between the virtual camera center and the vertex position projected onto the cone center line. To further improve spatial perception, the colorization of the anatomical structures in the in situ visualization was based on the distance from the model vertices to the endoscope tip:

(7) $$C_{final}=\left(1-\frac{d_{vertex}}{d_{far}}\right)\cdot C_{near}+\frac{d_{vertex}}{d_{far}}\cdot C_{far}$$

**Figure 3:**
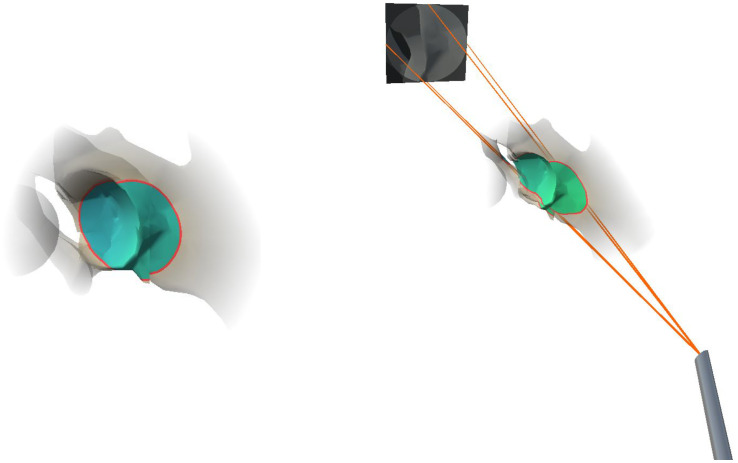
Visualization of the augmented target anatomy (**left**). The red outline marks the anatomical area in focus, while colorization encodes the distance to the endoscope. The anatomical visualization is combined with the frustum visualization (**right**).

$C_{near}$ and $C_{far}$ are the RGB color values associated with the camera center and the far clipping plane, and were set to (0.05, 0.9, 0.05) and (0.05, 0.05, 0.9), respectively. We chose the RGB color values of $C_{near}$ and $C_{far}$ to interpolate the color of the bone vertices in the frustum between a blue color tone at the far clipping plane and a green color tone at the near clipping plane. The final visualization of the anatomical area and the integration of the endoscope frustum is illustrated in Figure 3. All visualizations were implemented in a fragment shader program on the GPU.

## 4. Experiments and Results

In the following sections, we describe the user study setup (Section 4.1), the experimental variables (Section 4.2) and the hypotheses (Section 4.3). Afterwards, the information about the participants (Section 4.4) and the procedure (Section 4.5) is specified. We conclude this section by reporting our findings from the quantitative assessments and survey analysis (Section 4.6).

### 4.1. User Study Setup

**Figure 4:**
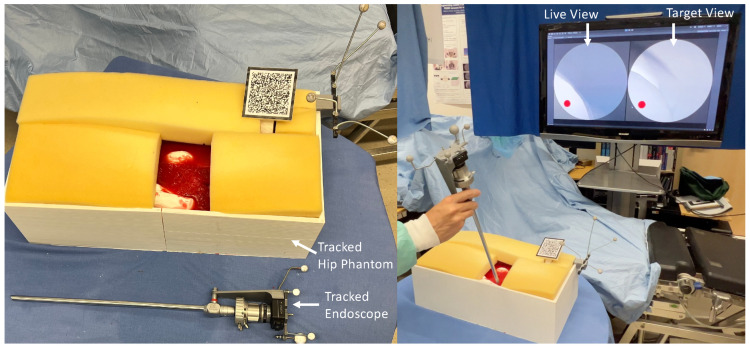
The simulation setup for our user study consisted of a tracked hip phantom and a tracked angled endoscope. The live view of the virtual endoscope, as well as the target view, were displayed on an external monitor.

**Figure 5:**
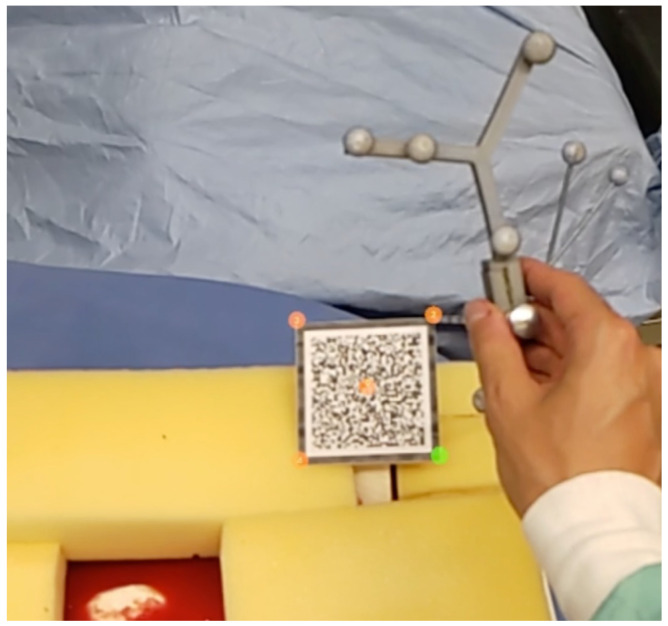
Calibration procedure between HMD and NDI tracking space.

For our physical phantom setup, we segmented the hip and femur bones from the open-source 3D human anatomy model: z-anatomy (https://www.z-anatomy.com/ (accessed on 5 October 2022)), and designed the marker attachment to the bone for localization. To evaluate if our proposed system provides adequate guidance and could improve the placement of an angled endoscope for hip arthroscopy, we developed a solution using Unity3D (Unity Technologies, San Francisco, CA, USA), which was deployed to an optical see-through HMD (OST-HMD), the Microsoft HoloLens 2 (Microsoft, Redmond, WA, USA). To evaluate the effectiveness of our system, we conducted a user study in which participants were asked to navigate an angled endoscope to predefined target locations for hip arthroscopy. For this reason, we designed a study setup consisting of a hip phantom model, an angled endoscope and an external monitor, illustrated in Figure 4. The phantom bone models, as well as the endoscope, were tracked with an external tracking system ART Dtrack2 (Advanced Realtime Tracking, Weilheim, Oberbayern, Germany). Furthermore, a Vuforia visual marker (PTC, Boston, MA, USA) was attached to the hip phantom to facilitate the correct augmentation of the virtual objects in the HoloLens 2. This initial calibration between the HMD coordinate system and the ART-tracking system was performed by a calibration procedure, as depicted in Figure 5. Once the tracking system was setup, the calibration procedure for every participant took no longer than two minutes.

**Figure 6:**
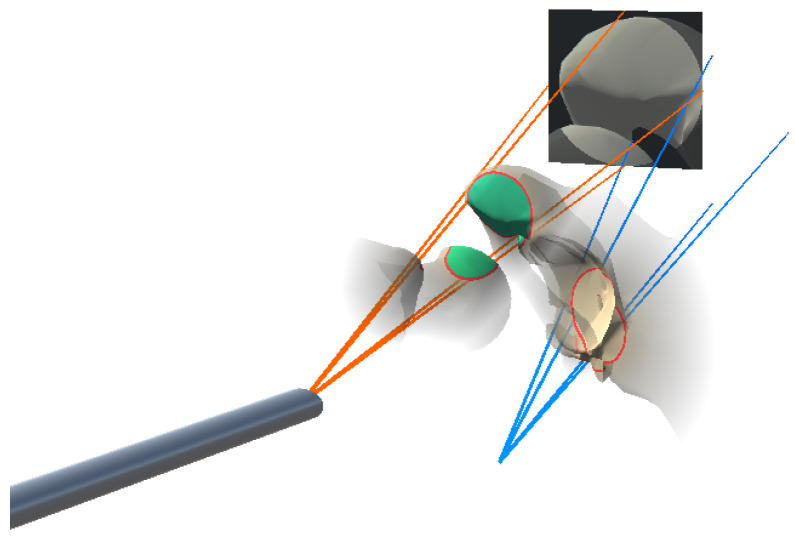
The orange frustum and depth-based colorization of the anatomical focus area show the current endoscopic pose, while the blue frustum and the in situ visualization without colorization shows the desired target pose.

The external monitor illustrated in Figure 4 shows both the virtual endoscopic view and the view from the desired target pose. Participants were asked to navigate the endoscope to find target areas given the endoscopic view of the target area. As shown in Figure 4, the target view, as well as the endoscopic live view, was displayed on an external monitor. For the baseline task of navigating without an AR system, the users were asked to position the endoscope using only the streamed endoscopic live view on the external monitor by aligning it with the target view. However, during the AR-supported positioning, users could see the virtual endoscopic frustum and in situ visualization described in Section 3. In addition, we augmented the frustum of the target endoscope pose, as well as the focus outline of the target pose on the anatomy, as illustrated in Figure 6.

### 4.2. Experimental Variables and Measures

The distance and orientation accuracy of the alignment, as well as the time to completion, were selected as dependent variables. The distance error was computed as the Euclidean distance between the gravity centers of the real and virtual objects, and the orientation errors were calculated as the angle, in degrees, between the objects using an axis-angle representation. Finally, the time to completion was considered as the time that elapsed since the first change in the pose of the virtual object until alignment confirmation from the user. In addition, we collected qualitative scores using a 7-point Likert scale adapted from the NASA task load index questionnaire (TLX) [21].

### 4.3. Hypotheses

Our principal assumption is that the use of our AR system would benefit the overall alignment accuracy without significantly affecting the time required to complete the alignment task. Thus, we present the following hypotheses:

**Hypothese** **1.** *Participants can achieve significantly better alignment scores when using AR visualization.*

**Hypothese** **2.** *Participants can complete the alignment task with significantly less time when using AR visualization.*

**Hypothese** **3.** *Participants can complete the alignment task with significantly less mental effort when using AR visualization.*

### 4.4. Participants

A total of seven users were recruited using mailing lists and campus announcements. The participants were aged between 25 and 29, with a mean age of 27.0±2.2 years. The participants had different levels of experience with augmented reality systems, ranging between zero and eight years, with an average of 3.7 years of experience. Participation in the study was voluntary and could be aborted at any time and performed in accordance with the Declaration of Helsinki. Symptoms of, or exposure to, COVID-19 were a hard exclusion criterion. All data collected from the study were anonymized.

### 4.5. Procedure

The participants were informed about the study procedure and were provided with a consent form. Upon completion of this form, participants were asked to perform a set of visual tests, including the Ishihara, Landolt, and stereo tests, to ensure correct or corrected-to-normal vision. Our exclusion criteria included color vision deficiency, impaired stereopsis (>140${}^{\circ }$ angle of stereopsis at 40 cm) or a visual acuity below 63% (20/32), and all participants were eligible for the study. Before starting the study, participants were provided with the HMDs and asked to perform a device calibration to adjust the interpupillary distance (IPD), after which they received a short introduction to augmented reality and HMD. After completion of the introduction, participants were asked to stand in front of the phantom and a big screen monitor. Participants were presented with 10 alignment tasks, split into 5 non-AR tasks and 5 tasks with our AR system. The alignment order was pseudo-randomly assigned using a Latin squared matrix. The poses of the target endoscope were predefined by a medical expert to ensure surgical relevance. Upon successful completion of the alignment tasks, participants were asked to complete a Raw-TLX questionnaire.

### 4.6. Results

In the following sections, we report the results of our user study. We collected quantitative measurements of the targeting accuracy with our AR system compared to the baseline method, as well as results from the survey analysis.

#### 4.6.1. Accuracy

**Figure 7:**
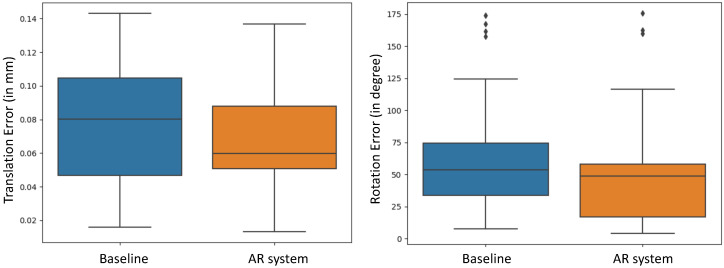
The pose error of the alignment task for both baseline and AR-aided endoscope positioning. We separately evaluated the position and rotation error of the poses.

Comparing the poses of the endoscope after performing the positioning task to the respective ground-truth endoscope poses, we individually evaluated the positional error of the endoscope tip and the rotational error. As shown in Figure 7, we observed a mean translational error of 7.9±3.7 cm for the baseline positioning and a mean error of 6.6±3.3 cm for AR-aided positioning. The average rotational error during baseline positioning was estimated to be 65.2±44.5 degree, while, for the AR-aided positioning, we estimated an average error of 51.3±45.4 degree error. These results suggest a trend towards higher accuracy when using the AR system compared to the baseline method. To further evaluate our findings, we conducted a two-sample t-test. The threshold for statistical significance was considered as *p* = 0.05. The results determined that the improvement in alignment accuracy in both translation (*p* = 0.1202) and orientation (*p* = 0.2056) had no statistical significance with our AR system in the conducted study.

#### 4.6.2. Temporal Performance

**Figure 8:**
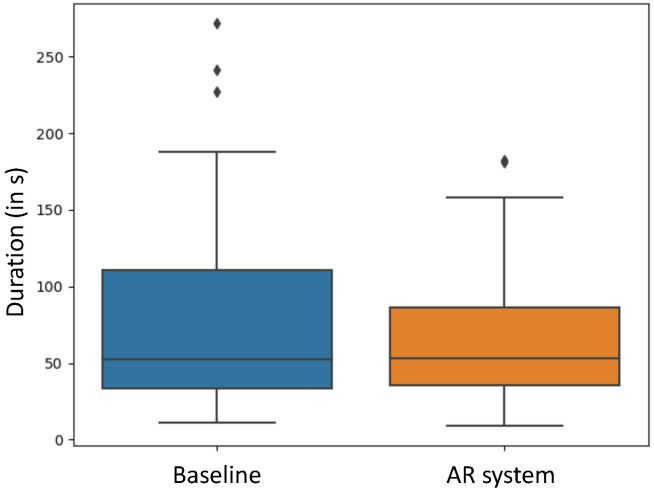
The duration of the alignment task per trial, comparing baseline and AR-guided positioning.

As shown in Figure 8, performing the positioning tasks took, on average, 83±70 s for the baseline positioning, while, for the AR-guided system, an average elapsed time of 70±48 s was measured. In addition, the t-test did not reveal statistical significance for the time to complete the alignment task (*p* = 0.3466).

#### 4.6.3. Subjective Ratings

**Figure 9:**
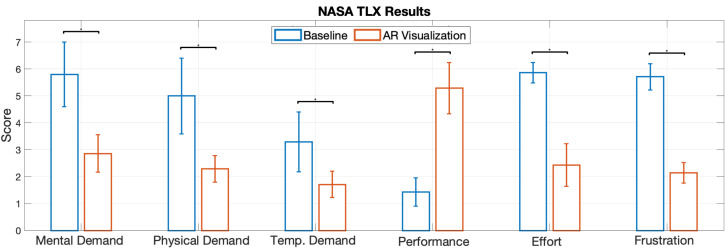
Subjective task load rating for the alignment.

We used NASA-TLX to subjectively evaluate the task load of the alignment task with and without AR visualization, and the results are shown in Figure 9. As demonstrated in the chart, the mental demand, physical demand, temporal demand, and effort level were perceived to be lower in the study with AR. In addition, the performance of alignment was perceived to be better with AR visualization, considering the average value. Considering the non-normal distribution of the task load index data, we applied a one-sided Wilcoxon signed rank test with α=0.05 for the subjective measures. The statistical results showed that the mental demand (*p* = 0.0156), physical demand (*p* = 0.0156), temporal demand (*p* = 0.0156), the effort level (*p* = 0.0078) and the frustration (*p* = 0.0078) were significantly reduced, while the performance significantly increased (*p* = 0.0078).

## 5. Discussion

Results from a user study comparing these interactive schemes using AR HMDs shows that the AR-guided solution has advantages in terms of usability, related to a decrease in physical demand, mental effort and frustration. Overall, participants achieved lower distance and orientation errors when using the proposed AR solution compared to the Baseline. However, the statistical significance could not be shown to support our hypothesis H1. We noticed that there are a few large orientation errors centered around 180 degrees, while the translational errors associated with the data are small in both cases. This shows that it is still very challenging for users to find the correct orientation with an angled endoscope, even with the help of AR, which might be related to the steep learning curve associated with the alignment of an angled endoscope. Nevertheless, on average, participants in the study took less time to complete the alignment tasks using an AR-aided endoscope positioning compared to the baseline. However, similar to the pose error, statistical significance could not be proven; thus, hypothesis H2 could not be supported. While we believe this trend could become more significant with the addition of more users, further studies need to be conducted in this regard. In terms of the subjective measures, the scores reported by the participants show significant differences in every category between the baseline and our proposed AR solution. This implies that the usability of our system significantly improves the Baseline method, supporting the hypothesis H3. This finding further suggests that, compared to the Baseline method, the cognitive load used while positioning an angled endoscope could be reduced when using our AR system.

Despite the proposed AR solution achieving significantly better results in a qualitative evaluation, with similar quantitative results to the baseline method, there are still a few limitations. The setup for the tracking system and calibration before the procedure is complicated and time-consuming compared to the baseline method. While we tried to mitigate potential third-variable biases that could have impacted the user study outcome, some limitations are inherent to the study design. First, the tracking space to HMD space calibration is subject to calibration errors, and thus can affect the user alignment. Recent works have explored the possibility of accurately tracking IR makers using sensors on HMD [22,23]. Martin-Gomez et al. [24] report comparable tracking accuracy to the external tracking system. Without the need to calibrate an external tracking system, the final propagated error from the pivot calibration of the point tool to the manual point set registration could be reduced in the system. Additionally, the rendering of the hip and femur model could not provide sufficient realism due to a lack of stimulations of the flesh, ligament, etc., which are present in the real anatomy. Fewer landmarks are visible to the users in the study, and this affected the alignment outcome. One aspect that is necessary for the integration of such an AR system into surgical setups is the registration of the pre-operative hip model to the patient anatomy. To incorporate this into our setup, available methods of CT-to-X-ray registration [25,26] could be employed to enable augmentation of the bone model in the patient. In future works, we could also use imaging to integrate such a method into a robotic setup for the improved guidance of endoscopic tools.

## 6. Conclusions

In this work, we proposed a novel method for AR assistance during the positioning of an angled endoscope for hip arthroscopy. An HMD-based application was developed and evaluated with a mock 3D-printed setup. Our system integrates features of frustum visualization, augmentation of the anatomical focus area and distance-based colorization. We conducted a user study, in which we compared the endoscope positioning with our AR system to baseline positioning without additional support. The results from our user study suggest that the average endoscope positioning accuracy could be improved using our AR system. Furthermore, the speed at which endoscope positioning was performed could be increased. In addition, statistical significance was demonstrated in terms of usability, indicating a lower cognitive load when aligning an angled endoscope with our AR system compared to baseline positioning. We believe that such a method could improve the overall operation quality, decrease the cognitive effort of aligning an angled endoscope, and might reduce the required X-ray dose during surgery.

## Data Availability

All the reported data can be found at https://nextcloud.in.tum.de/index.php/s/dGcAc7kBjKddkzD (accessed on 6 October 2022).
